# DNA methylation in the promoter region of the *p16* (*CDKN2/MTS-1/INK4A*) gene in human breast tumours

**DOI:** 10.1038/sj.bjc.6690041

**Published:** 1999-01

**Authors:** D M Woodcock, M E Linsenmeyer, J P Doherty, W D Warren

**Affiliations:** Peter MacCallum Cancer Institute, Melbourne, Victoria, Australia

**Keywords:** breast tumour, *p16*, CpG island, DNA methylation, genomic sequencing, SP1 sites

## Abstract

The *p16 (CDKN2/MTS-1/INK4A)* gene is one of several tumour-suppressor genes that have been shown to be inactivated by DNA methylation in various human cancers including breast tumours. We have used bisulphite genomic sequencing to examine the detailed sequence specificity of DNA methylation in the CpG island promoter/exon 1 region in the *p16* gene in DNA from a series of human breast cancer specimens and normal human breast tissue (from reductive mammaplasty). The *p16* region examined was unmethylated in the four normal human breast specimens and in four out of nine breast tumours. In the other five independent breast tumour specimens, a uniform pattern of DNA methylation was observed. Of the nine major sites of DNA methylation in the amplified region from these tumour DNAs, four were in non-CG sequences. This unusual concentration of non-CG methylation sites was not a general phenomenon present throughout the genome of these tumour cells because the methylated CpG island regions of interspersed L1 repeats had a pattern of (almost exclusively) CG methylation similar to that found in normal breast tissue DNA and in DNA from tumours with unmethylated *p16* genes. These data suggest that DNA methylation of the *p16* gene in some breast tumours could be the result of an active process that generates a discrete methylation pattern and, hence, could ultimately be amenable to theraputic manipulation. © 1999 Cancer Research Campaign


*British Journal of Cancer* (1999) **79**(2), 251–256
© 1999 Cancer Research Campaign

### DNA methylation in the promoter region of the *p16* (*CDKN2/MTS-1/INK4A)* gene in human breast tumours

**DM Woodcock, ME Linsenmeyer, JP Doherty and WD Warren**

Peter MacCallum Cancer Institute, Melbourne, Victoria, Australia

**Summary** The *p16 (CDKN2/MTS-1/INK4A)* gene is one of several tumour-suppressor genes that have been shown to be inactivated by DNA methylation in various human cancers including breast tumours. We have used bisulphite genomic sequencing to examine the detailed sequence specificity of DNA methylation in the CpG island promoter/exon 1 region in the *p16* gene in DNA from a series of human breast cancer specimens and normal human breast tissue (from reductive mammaplasty). The *p16* region examined was unmethylated in the four normal human breast specimens and in four out of nine breast tumours. In the other five independent breast tumour specimens, a uniform pattern of DNA methylation was observed. Of the nine major sites of DNA methylation in the amplified region from these tumour DNAs, four were in non-CG sequences. This unusual concentration of non-CG methylation sites was not a general phenomenon present throughout the genome of these tumour cells because the methylated CpG island regions of interspersed L1 repeats had a pattern of (almost exclusively) CG methylation similar to that found in normal breast tissue DNA and in DNA from tumours with unmethylated *p16* genes. These data suggest that DNA methylation of the *p16* gene in some breast tumours could be the result of an active process that generates a discrete methylation pattern and, hence, could ultimately be amenable to theraputic manipulation.

**Keywords:** breast tumour; *p16*; CpG island; DNA methylation; genomic sequencing; SP1 sites

Alterations in the function of multiple genes are required for the full development of oncogenic phenotype in humans and other mammals (Knudson, 1971; Vogelstein et al, 1987). In the case of tumour-suppressor genes, it is now clear that loss of function can not only occur through allelic loss or mutation, but also through loss of function mediated by DNA methylation (Baylin, 1992; Szyf, 1994; Merlo et al, 1995). The *p16* gene (*CDKN2*/*MTS-1*/*INK4A*) is one such gene. This gene acts to inactivate CDK4 and CDK6 cyclin-dependent kinases and, hence, controls the entry of cells from $G_1$ to S-phase (Kamb, 1995; Hara et al, 1996). As such, it is a prime candidate as a tumour-suppressor gene. However, whereas this gene was found to be subject to frequent homozygous deletion in tumours, point mutations were not commonly detected. Consequently, the role of *p16* as a tumour suppressor was questioned until it was shown that the *p16* gene was also frequently inactivated through a process involving DNA methylation without point mutation (Merlo et al, 1995; Herman et al, 1996*a*).

In human breast tumours, it has been reported that > 30% of primary tumours and many tumour-derived cell lines have inactive methylated copies of the *p16* gene (as assayed at only a few sites using methylation-sensitive restriction endonucleases) (Merlo et al, 1995). Herman et al (1996*b*) have further shown by bisulphite genomic sequencing that in a tumour cell line (H157) a region of the *p16* gene becomes completely methylated at all CG dinucleotides. Extrapolating from this finding, they developed an assay that will detect a very small proportion of tumour cells containing methylation at all CG sites as a sensitive method of detecting minimal residual disease. This method uses polymerase chain reaction (PCR) primers that amplify specifically from the fully CG-methylated form of the gene after bisulphite conversion of unmethylated cytosines to uracils (Frommer et al, 1992). Using this assay, they have demonstrated the presence in a series of human tumour-derived cell lines and in some human tumours of apparently fully CG-methylated forms of *p16* and other genes (Herman et al, 1996*b*).

Here, we report the results of genomic sequencing from DNA from a series of human breast samples, both normal and tumour, and demonstrate a common specific pattern of tumour-related DNA methylation in a number of independent human breast tumours. Rather than a non-specific methylation at CG dinucleotides in this gene, this methylation has characteristics that could better be explained as the product of a gene-specific alteration in the local secondary structure of the promoter/exon1 region that transforms it into a highly efficient substrate for asymmetric de novo methylation.

### MATERIALS AND METHODS

Human tissue specimens were portions of samples provided for routine pathology assessment that were in excess of the requirements for that purpose and were obtained with the approval of the relevant ethics committees. All samples were stored at –70°C until DNA was extracted by the guanidine isothiocyanate/caesium chloride method for the isolation of both RNA and DNA (Chirgwin et al, 1979), and further purified by extraction with phenol/chloroform. For genomic sequencing of sites of DNA methylation, 5 μg of genomic DNA was modified with metabisulphite after alkali denaturation using five or six cycles of 94°C for 3 min, 55°C for 57 min and subsequently desulphonated as described previously

*Received 4 November 1997*
*Revised 16 April 1998*
*Accepted 6 May 1998*

*Correspondence to:* DM Woodcock, Peter MacCallum Cancer Institute, Locked Bag No. 1, A'Beckett Street, Melbourne, Victoria 8006, Australia

**Figure 1** Region amplified from the promoter/exon1 region of the human *p16* gene. The sequence is derived from two overlapping GenBank entries: HSPCDNK2 and HSPCDK1. The PCR primer regions are shown with the dotted underlining. This region contains two direct repeats of 24 bp and 9 bp, with the first of each pair shown with single underlining and the second of each pair with double underlining. SP1 core consensus sequences (Minth and Dixon, 1990; Thiesen and Bach, 1990; Merchant et al, 1991) are shown in italics and the ATG initiation codon as bold capitals. The predominant methylation pattern contains all of the capitalized Cs between the primers except those at bases 204 and 272. The minor methylation pattern found in ts34 only consisted of the Cs at bases 204, 244 and 272

(Woodcock et al, 1997). Modifications included an internal control and a linearized plasmid DNA containing an human CpG island insert (from the 5′ end of an L1 repeat) as used in previous studies using bisulphite genomic sequencing (Woodcock et al, 1997, 1998). Only modifications in which clones amplified from this control sequence were found to contain < 0.2% residual cytosines (i.e. effectively completely modified) were used for amplification for genomic sequencing of methylation sites. In all instances, each set of clones are derived from multiple template modification and amplification reactions done on different days. For amplification of the *p16* sequences, the primers used were 5′-AGGATTTGAGGGA(C/T)AGGG-3′ and 5′-CCAATTCCCCT(G/A)CAAACTTC-3′. These corresponded to bases 1059–1073 of the human p16 promoter/exon 1 CpG island region (GenBank:HSPRCDNK2) and the complement of bases 308–327 from the overlapping GenBank entry for human p16 exon 1 (HSPCDK1). PCR products were cloned into pGEM-T vector (Promega) and transformed into highly electrocompetent DH12S *Escherichia coli* host cells. Random white colonies were screened for the correctly sized insert (70–100% of those screened) and sequenced with the forward and reverse universal primers using the ABI 377 system using dye terminator chemistry (Applied Biosystems). Genomic sequencing from the 5′ hypermethylated CpG island region of human L1 elements was performed as described previously (Woodcock et al, 1997).

### RESULTS

In the bisulphite method for the genomic sequencing of sites of DNA methylation, unmethylated cytosines are converted to uracils and, through subsequent PCR amplification, to thymines (Frommer et al, 1992). After this process, the two strands of any region of duplex DNA are no longer complementary (unless all cytosines in the sequence are methylated). Consequently, different primers are required to amplify the ‘top’ and ‘bottom’ strands of any DNA sequence after bisulphite modification.

However, if unwarranted a priori assumptions as to methylation status of any DNA sequence are used in primer design, it is possible [and even likely (Herman et al, 1996*b*)] that PCR will only amplify from some subset of DNA strands whose methylation pattern may not necessarily reflect the methylation of most DNA from that sample. In this study, PCR primers for the *p16* gene were designed so that they would be able to prime amplification from both modified and unmodified (native) forms of the target sequence, irrespective of methylation status of any or all cytosines in the primer sequences (Woodcock et al, 1997, 1998). This was achieved by choosing a site for the forward primer that was rich in guanines and contained the minimum number of cytosines (and also lacked simple sequence tracts). Any cytosine in the PCR forward primers was synthesized as a degenerate site (C or T), irrespective of dinucleotide context. For the reverse primer complementary to the strand amplified, this strategy for primer design results in a primer rich in cytosines and with any guanines as a degenerate G or A. No assumptions were made that methylation was only in CG dinucleotides because it now appears that, in some instances, mammalian cells can have methylation at sites other than CG dinucleotides (Woodcock et al, 1987, 1997; Toth et al, 1990; Clark et al, 1995; Snibson et al, 1995).

The sequence of the region amplified from the *p16* gene is shown in Figure 1. This figure illustrates the positions of sequence features such as the ATG initiation codon for the main transcript from the *p16* gene (Mao et al, 1995), the two pairs of tandem repeats, and three SP1 core consensus sequences (Minth and Dixon, 1990; Thiesen and Bach, 1990; Merchant et al, 1991). Using native (not bisulphite-modified) normal human DNA as a template, PCR amplification with these primers generated clones from the *p16* gene that were identical in sequence to the corresponding GenBank entries (Figure 2).

DNA from four normal human breast samples (from reductive mammaplasty) and from nine breast tumours were modified with bisulphite and the modified form of DNA strands from the *p16* gene amplified with PCR primers as described above. A total of 137 independent clones derived from the tumour DNAs and 32 clones from the normal breast DNAs were sequenced. Only clones from modification reactions in which DNA derived from the internal control sequence had been effectively converted were analysed. All sets of clones are derived from two or more independent modification and amplification reactions

Clones of the PCR product from bisulphite-modified normal breast DNAs showed this region was essentially devoid of methylation except for low levels of residual cytosines in apparently random sites (Figure 2). This was also the case for clones from four of the tumour sample DNAs. The *p16* sequences amplified from human embryonic fibroblast DNA were also unmethylated (DW, unpublished data). However, clones from five of the independent tumour samples showed a consistent pattern of methylation principally in the same set of nine sites (Figure 2). The regions of the tumour-derived *p16* clones aligned in this figure contain eight of the nine consistently methylated sites, the other site of methylation being at base no. 356 that is immediately adjacent to the reverse primer sequence (Figure 1).

All the tumour samples contained unmethylated clones, as expected, as any surgical sample is likely to contain a mixture of normal and tumour cells, with the relative proportion of methylated clones possibly reflecting the proportion of tumour cells in the specimen. In the tumour samples ts18, ts23, ts24, ts34 and ts43, these sites were coordinately methylated in 11%, 24%, 10%, 18% and 17% of clones, respectively, with all but one of the clones having all nine of these residual cytosines. There was also another pattern of coordinated methylation observed in 11% of clones from ts34 (methylation at base numbers 204, 244 and 272) that

© Cancer Research Campaign 1999

| Sample source | Total clones in set | Percentage of each type | Sequence context |
|---|---|---|---|
| (base number) | | | 174 210 229 278 |
| GenBank entry | | | CAGCATGGAGCCTTCGGCTGACTGGCTGGCCACGGCC~/~GAGGAGGTGCGGGCGCTGCTGGAGGCGGGGGCGCTGCCCAACGCACCGAA |
| Unmodified normal DNA | | | CAGCATGGAGCCTTCGGCTGACTGGCTGGCCACGGCC~/~GAGGAGGTGCGGGCGCTGCTGGAGGCGGGGGCGCTGCCCAACGCACCGAA |
| Fully modified unmethylated | | | TAGTATGGAGTTTTTGGTTGATTGGTTGGTTATGGTT~/~GAGGAGGTGTGGGTGTTGTTGGAGGTGGGGGTGTTGTTTTTTGTATTGAA |
| nb4 | 11 | 73 | -------------------------------------~/~-------------------------------------------------- |
| | | 9 | ------C------------------------------~/~-------------------------------------------------- |
| | | 9 | ------------------------------------C~/~---------------C----------------------C----------- |
| | | 9 | -------------------------------------~/~-----------------------------------------C----C--- |
| nb2 | 11 | 100 | -------------------------------------~/~-------------------------------------------------- |
| nb5 | 8 | 100 | -------------------------------------~/~-------------------------------------------------- |
| nb1 | 4 | 100 | -------------------------------------~/~-------------------------------------------------- |
| **ts23** | 34 | 62 | -------------------------------------~/~-------------------------------------------------- |
| | | **24** | **----------C----------C---------------~/~---------C---C-C--C------C-----C------------------** |
| | | 3 | ---C---------------------------------~/~---------------------------------C---------------- |
| | | 3 | ---C---------------------------------~/~-------------------------------------------------- |
| | | 3 | -------------------------------------~/~------------------C----A-------------------------- |
| | | 3 | -----------------------------------C-~/~-----------------------------------------C-------- |
| | | 3 | -------------------------------------~/~-----------------------------------------C-------- |
| **ts34** | 28 | 61 | -------------------------------------~/~-------------------------------------------------- |
| | | **18** | **----------C----------C---------------~/~---------C---C-C--C------C-----C------------------** |
| | | 11 | ------------------------------C------~/~---------------C---------------------------C------ |
| | | 3.6 | ---C---------------------------------~/~-------------------------------------------------- |
| | | 3.6 | -----------------------------------C-~/~-----------------------------------------C-------- |
| | | 3.6 | C------------------------------------~/~-------------------------------------------------- |
| **ts43** | 18 | 78 | -------------------------------------~/~-------------------------------------------------- |
| | | **17** | **----------C----------C---------------~/~---------C---C-C--C------C-----C------------------** |
| | | 5 | -------------------------------------~/~----------------------------------------G--------- |
| **ts24** | 10 | 90 | -------------------------------------~/~-------------------------------------------------- |
| | | **10** | **----------C----------C---------------~/~---------C---C-C--C------C-----C------------------** |
| **ts18** | 9 | 89 | -------------------------------------~/~-------------------------------------------------- |
| | | **11** | **----------C----------C---------------~/~---------C---C-C--C------C-----C------------------** |
| ts30 | 12 | 91 | -------------------------------------~/~-------------------------------------------------- |
| | | 9 | ------------------------A--AA--------~/~------------------------A------------------------- |
| ts21 | 10 | 70 | -------------------------------------~/~-------------------------------------------------- |
| | | 10 | ---C---------------------------------~/~-------------------------------------------------- |
| | | 10 | -------------------------------------~/~---------------------------------C-------C----C--- |
| | | 10 | -------------------------------------~/~-----------------------------------------C-------- |
| ts32 | 10 | 80 | -------------------------------------~/~-------------------------------------------------- |
| | | 10 | -----------------------------------C-~/~-----------------------------------------C-------- |
| | | 10 | -------------------------------------~/~----------------------------------------------C--- |
| ts41 | 7 | 72 | -------------------------------------~/~-------------------------------------------------- |
| | | 14 | ---C---------------------------------~/~-------------------------------------------------- |
| | | 14 | -------------------------------------~/~--------------------A----------------------------- |

**Figure 2** Alignment of *p16* sequence data from PCR products from bisulphite-modified normal and tumour DNAs in the region of bases 174–210 and 229–278. These regions contain eight out of the nine consistently methylated sites observed in these clones. Only very low levels of random methylation were detected between bases 1 and 173, 211 and 228, and 279 and 355. The genomic sequence from the GenBank entries (Figure 1) is given across the top. An identical sequence was found in clones from the human *p16* gene amplified using these primers from unmodified DNA from non-transformed human embryonic fibroblasts (second line). Below this is the sequence as it would appear if every C were converted to a T (complete conversion of a completely unmethylated form of the sequence). Underneath this is the aligned compilation from all of the clones from modified normal breast DNAs ('nb1' to 'nb4') and from tumour DNAs ('ts18' through to 'ts43'). A dash indicates identity with the sequence expected for the full bisulphite conversion of the unmethylated form of the *p16* sequence. The clones from each tumour DNA are grouped with respect to sequence identity within that group. The total clones sequenced from each tumour DNA are shown together with the per cent of clones in each group showing sequence identity within these two regions. Four of the tumour-derived specimens were basically unmethylated as were those from normal breast DNAs (i.e. low levels of random residual cytosines). Clones from the other five tumours were either unmethylated (presumably derived from normal cells in the surgical sample) or methylated with a consistent pattern of residual cytosines. Five other clones derived from four different template DNAs were excluded from this analysis. These contained regions with strings of adenine substitutions reminiscent of PCR amplification from non-instructional lesions or abasic sites (Strauss, 1991). Complete alignments of all clones are available from the authors on request was not observed in any of the clones from the other modified DNAs. In these two distinct methylation patterns observed in the ts34 clones, base 244 was the only methylated site common to both patterns (Figure 2). In the remaining sets of tumour-derived *p16* clones that did not exhibit discrete methylation patterns, there was a slightly higher proportion of (apparently random) methylated bases present overall than in clones from normal breast DNAs. However, methylation levels were somewhat lower overall than in the tumours with discrete methylation.

In the tumour-derived clones exhibiting discrete methylation patterns, a high proportion of residual cytosines (methylation sites) were on non-CG contexts (Figures 1 and 2). In the predominant tumour methylation pattern, five out of nine sites in the region amplified were in $^{m}$CG dinucleotides, but three out of nine were in $^{m}$CTG contexts and one in what would have been $^{m}$CCTTCG. In the secondary methylation pattern found in some clones from ts34, the original sequence contexts would have been $^{m}$CACG, $^{m}$CTG and $^{m}$CACCG. The few random residual cytosines that were observed in clones from normal breast DNAs were almost exclusively in the context of CG dinucleotides (not illustrated).

To test whether this unusually high proportion of non-CG methylation observed in the *p16* gene from these tumour DNAs

© Cancer Research Campaign 1999

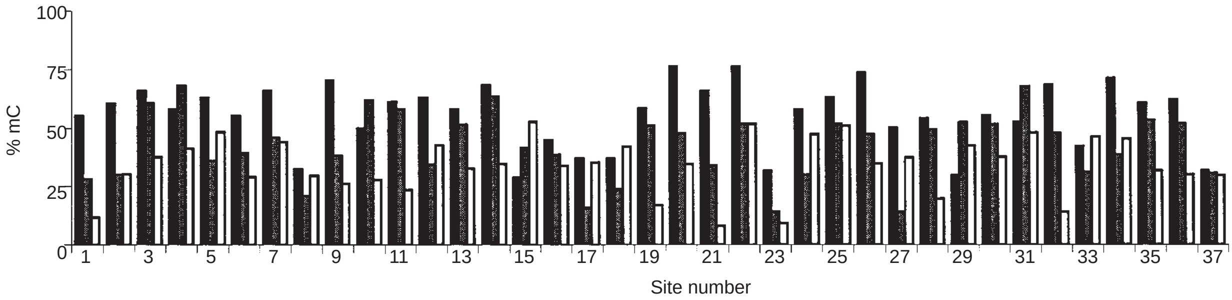

**Figure 3** Proportion of clones with residual cytosines in each of the consistently methylated sites from the methylated CpG island region from human *L1* dispersed repeats. Values are given as percentage of the total clones with cytosines averaged over all the clones from normal breast DNAs (■), the clones from tumours with methylated *p16* gene (▩), and the clones from tumour DNAs where *p16* exhibited low levels of random methylation (as in normal breast) (□). The *x*-axis represents the numbering from 5′ to 3′ of the consistently methylated CG sites. Sets of full sequence data relating site number to base number are available on request might reflect some widespread deregulation (or misregulation) of the methylation processes, DNA sequences from the methylated CpG island region of human *L1* dispersed repeat elements (Woodcock et al, 1997) were amplified, cloned and sequenced. This allowed the sampling from multiple dispersed sites of methylation through the genome rather than from just a single site as would be the case with a unique protein coding sequence. In clones from these sequences amplified from bisulphite-modified DNAs from normal breast samples, a total of 37 consistently methylated sites were observed in a 460-bp region with an average 55.5% residual cytosines at these sites. (These are observed frequencies of cytosines only with no correction having been made for sequence variations within the repeat sequence family.)

*L1* clones from two tumour DNAs with basically unmethylated *p16* genes (ts21 and ts30) had 34.0% residual cytosines present on average at these sites, whereas the average for the clones from the tumours with methylated *p16* was 42.5% residual cytosines. The methylation levels in dispersed repeats are likely to reflect average methylation levels in total genomic DNA, and are consistent with previous observations that tumours frequently have lower total genomic methylation levels which may be accompanied by increased methylation in some normally unmethylated CpG island regions (such as the *p16* promoter) (reviewed by Szyf, 1994). The relative levels of methylated cytosines at each of these consistently methylated sites in *L1* elements from DNAs from normal and tumour-derived DNAs (with and without *p16* methylation) are shown in Figure 3. All 37 of the consistently methylated sites in clones from the *L1* repeats from both normal and tumour DNAs were in CG dinucleotides. However, there were some methylated non-CG sites observed. The occurrence of sites of non-CG methylation, however, was suggestive of individual polymorphisms in the control of epigenetic modification. For example, seven out of nine clones from one normal breast sample (nb1) had a $^{m}$C$^{m}$CA sequence. This site was also methylated in four out of nine and one out of ten clones from two other sets of normal breast-derived clones (nb2 and nb5 respectively), but absent from clones from the fourth normal breast sample and also from all tumour-derived clones. Also, the tumour DNA ts30 in which the *p16* gene was unmethylated had two $^{m}$CAG sites, one $^{m}$CTT and one $^{m}$CATTAG site in up to one-third of clones (not illustrated). In addition, at site no. 15 in ts30 clones, 0 out of 12 clones had a residual cytosine at this CG dinucleotide. Rather, five of these clones had a residual cytosine as the prior base (i.e. originally $^{m}$CCG rather than C$^{m}$CG).

### DISCUSSION

Using genomic sequencing of DNAs from human clinical specimens, we have observed a common pattern of DNA methylation in the *p16* gene from a series of independent breast tumour samples. This methylation pattern is of unusual sequence specificity and contains a high proportion of methylation at asymmetric sites. We consider that this unusual methylation pattern is not an experimental artefact for the following reasons:

1. The PCR product from modifications of human genomic DNAs were not used unless clones from the control plasmid were effectively modified (< 0.2% residual cytosines excluding sites of endogenous *E. coli dcm* methylation) (Woodcock et al, 1997).
2. In clones from modified DNAs from four normal breast samples and from four of the breast tumours, the *p16* gene was essentially unmethylated (in a total of 72 independent clones comprising 33 from normal breast samples and 39 from four tumour samples). Thus, methylation at the specific set of sites observed in these five breast tumours is specific to DNA from these tumours and cannot represent some region that is intrinsically resistant to bisulphite modification.

Hence, it appears that the residual cytosines present in clones from modified DNAs from some human breast tumours represent a characteristic tumour-specific epigenetic modification to the DNA. Although clones from one of the tumour specimens showed evidence of a second pattern of DNA methylation in the *p16* gene, clones with the common predominant pattern of methylation were also present. (It is conceivable that these two methylation patterns represent two independent lines of oncogenic evolution in this particular tumour.)

Herman et al (1996*b*) have shown that, in some human tumour-derived cell lines and some clinical specimens, the *p16* gene can be present with complete methylation of CG dinucleotides. We cannot say from these data that the breast tumour DNAs used in this study do not contain some *p16* sequences with complete methylation at all of the CG dinucleotides. If copies of this gene with complete CG methylation were present at, for example, <10% of the frequency of sequences with the methylation pattern we have observed, we would have a low probability of recovering them, considering also that these clinical samples contain a mixture of normal cells. However, we have shown previously that

 © Cancer Research Campaign 1999

the PCR amplification and cloning methodology employed here does not bias the data through the selective recovery of clones from less methylated forms of the target sequence (Woodcock et al, 1997). It has also been shown that, during adaptation of cells to in vitro culture, many normally unmethylated sites in CpG island sequences can become methylated (Antequera et al, 1990). The observation of complete methylation in all CG dinucleotides in the *p16* gene from a human tumour-derived cell line is more likely to represent a later stage in the evolution of epigenetic modification of this CpG island rather than an artefact of in vitro culture. The ability to amplify from primary human tumours using PCR primers specific for the bisulphite-modified form of fully CG methylated *p16* gene argues strongly for the presence of some cells within primary tumours with this pattern of methylation (Herman et al, 1996*b*). The absence of clones with such a methylation pattern in this study could be due to such cells being in relatively low abundance. This would not be inconsistent with total methylation at CG dinucleotides representing an important and characteristic stage of tumour evolution, however an assessment of the minimal residual disease using an assay that identifies cells with total methylation of p16 would fail to detect tumour cells with other patterns of methylation.

The major concentration of methylated sites in the *p16* promoter/exon 1 region examined in this study is in a region containing two 9-bp direct repeats separated by 9 bp (Figure 1 and Figure 2). Overlapping with this central region and the 3′ repeat is a SP1 core consensus site (Minth and Dixon 1990; Thiesen and Bach, 1990; Merchant et al, 1991). Methylation in CG dinucleotides has been shown not to affect SP1 binding (Harrington et al, 1988). The presence of SP1 sites has also been shown to protect against de novo methylation and to induce demethylation adjacent to the SP1 site (Brandeis et al, 1994; Macleod et al, 1994; Silke et al, 1995). There are also two other SP1 consensus sites 70–100 bp upstream in two 24-bp tandem repeats with the more proximal repeat containing the *p16* ATG initiation codon plus the first of the tumour-specific methylation sites. In this instance, this concentration of SP1 binding sites has not prevented tumour-specific methylation in this *p16* CpG island region.

Methylation, particularly the high concentration of non-CG methylation observed in the *p16* gene region, was not part of some general non-specific epigenetic modification of the tumour genomes. When we sampled the sequence specificity of methylation in multiple regions of the normal and tumour breast DNAs using the CpG island region from the 5′ end of human *L1* repeats, methylation was consistently found at more sites than we observed previously in *L1* elements from non-transformed human embryonic fibroblasts (Woodcock et al, 1997). In this *L1* region, 29 consistently methylated sites were observed in embryonic fibroblast DNA as opposed to the 37 observed in this study with DNA from normal human breast. Also, for the *L1* elements from embryonic fibroblasts, 4 out of 29 consistently methylated positions were in non-CG sites, whereas, in this study, the consistently methylated sites were all in CG dinucleotides. However, some specific sites of non-CG methylation were present but these seemed to be specific to the individual, and may represent polymorphisms in genetic factors that determine epigenetic events. However, overall, there is no evidence for a general increase in non-CG methylation in the genome of those tumours that have a high proportion of non-CG methylation in their *p16* genes.

There is now evidence that mice (and presumably all other mammals) have more than one DNA methyltransferase gene. ES cells from complete knockouts of the know DNA methyltransferase gene retain residual DNA methylation as well as the ability to methylate sequences de novo (Lei et al, 1996; Tucker et al, 1996). This residual DNA methyltransferase gene has been suggested to represent the activity responsible for the wave of de novo methylation that occurs in the genome of the preimplantation embryo (Monk, 1990). However, the discrete methylation patterns observed in *p16* gene from tumour DNAs with their high proportion of methylation at non-CG sites is very different from the specificity of methylation in methyltransferase-knockout ES cells. This methylation is apparently totally CG-specific and it appears to be randomly positioned within the set of sites that are methylated in the normal adult mouse genome (Woodcock et al, 1998). Rather, the high proportion of non-CG methylation in the tumour *p16* genes is more likely to be related to the process whereby sites of CNG methylation in transfected plasmid DNAs can be maintained over many cell generations in mouse cells in culture (Clark et al, 1995).

For a distinct methylation pattern to be present, there must be some mechanism that efficiently restores this methylation in the daughter strands after each round of replication. Otherwise, such a methylation pattern would rapidly be lost. Maintenance of DNA methylation at any site in mammalian genomes in non-CG sites is conceptually inherently more difficult than that in CG dinucleotides in which there is the symmetrically placed $^{m}$CG template in the parental strand (Holliday and Pugh, 1975; Riggs, 1975). One possible exception is methylation in CNG sequences (as in plant DNAs) in which a parental template would be displaced by only one base, although this is not a documented function of the mammalian DNA methyltransferase. However, it has also been shown that distortions of the DNA duplex that promote the extrahelical extrusion of any cytosine base will render such a cytosine an efficient substrate for de novo methylation (Smith et al, 1992; Klimasauskas and Roberts, 1995; Laayoun and Smith, 1995). We suggest that one possible explanation for the discrete pattern of DNA methylation observed in the *p16* gene in breast tumour DNAs is that it is the result of sequence-specific protein binding that induces the DNA duplex in this region to be distorted, rendering certain specific bases efficient substrates for asymmetric de novo methylation. If this were the case, inactivation of some tumour-suppressor genes through DNA methylation would, thus, be an active process in the tumour rather than through random methylation at CG dinucleotides and, hence, be more amenable to ultimate therapeutic manipulation.

### ACKNOWLEDGEMENTS

We would like to thank Dr Michael Henderson for his help both in obtaining tumour samples and in organizing such collections from other sources. This study was partially financed by a grant to DMW from the National Health and Medical Research Council of Australia.

### REFERENCES

Antequera F, Boyes J and Bird A (1990) High levels of de novo methylation and altered chromatin structure at CpG islands in cell lines. *Cell* **62**: 503–514

Baylin SB (1992) Abnormal regional hypermethylation in cancer cells. *Aids Res Hum Retroviruses* **8**: 811–820

Brandeis M, Frank D, Keshet I, Siegfried Z, Mendelsohn M, Nemes A, Temper V, Razin A and Cedar H (1994) Sp1 elements protect a CpG island from de novo methylation. *Nature* **371**: 435–438

Chirgwin JM, Przybyla AE, MacDonald RJ and Rutter WJ (1979) Isolation of biologically active ribonucleic acid from sources enriched in ribonuclease. *Biochemistry* **18**: 5924–5929

Clark SJ, Harrison J and Frommer M (1995) CpNpG methylation in mammalian cells. *Nature Genet* **10**: 20–27

Frommer M, McDonald LE, Millar DS, Collis CM, Watt F, Grigg GW, Molloy PL and Paul CL (1992) A genomic sequencing protocol that yields a positive display of 5- methylcytosine residues in individual DNA strands. *Proc Natl Acad Sci USA* **89**: 1827–1831

Hara E, Smith R, Parry D, Tahara H, Stone S and Peters G (1996) Regulation of p16CDKN2 expression and its implications for cell immortalization and senescence. *Mol Cell Biol* **16**: 859–867

Harrington MA, Jones PA, Imagawa M and Karin M (1988) Cytosine methylation does not affect binding of transcription factor Sp1. *Proc Natl Acad Sci USA* **85**: 2066–2070

Herman JG, Jen J, Merlo A and Baylin SB (1996*a*) Hypermethylation-associated inactivation indicates a tumour suppressor role for p15INK4B1. *Cancer Res* **56**: 722–727

Herman JG, Graff JR, Myohanen S, Nelkin BD and Baylin SB (1996*b*) Methylation-specific PCR: a novel PCR assay for methylation status of CpG islands. *Proc Natl Acad Sci USA* **93**: 9821–9826

Holliday R and Pugh JE (1975) DNA modification mechanisms and gene activity during development. *Science* **187**: 226–232

Kamb A (1995) Cell cycle regulators and cancer. *Trends Genet* **11**: 136–140

Klimasauskas S and Roberts RJ (1995) Disruption of the target G–C base-pair by the HhaI methyltransferase. *Gene* **157**: 163–164

Knudson AG (1971) Mutation and cancer: statistical study of retinoblastoma. *Proc Natl Acad Sci USA* **68**: 820–823

Laayoun A and Smith SS (1995) Methylation of slipped duplexes, snapbacks and cruciforms by human DNA(cytosine-5)methyltransferase. *Nucleic Acids Res* **23**: 1584–1589

Lei H, Oh SP, Okano M, Jutterman R, Goss KA, Jaenisch R and Li E (1996) De novo DNA cytosine methyltransferase activities in mouse embryonic stem cells. *Development* **122**: 3195–3205

Macleod D, Charlton J, Mullins J and Bird AP (1994) Sp1 sites in the mouse aprt gene promoter are required to prevent methylation of the CpG island. *Genes Dev* **8**: 2282–2292

Mao L, Merlo A, Bedi G, Shapiro GI, Edwards CD, Rollins BJ and Sidransky D (1995) A novel p16INK4A transcript. *Cancer Res* **55**: 2995–2997

Merchant JL, Demediuk B and Brand SJ (1991) A GC-rich element confers epidermal growth factor responsiveness to transcription from the gastrin promoter. *Mol Cell Biol* **11**: 2686–2696

Merlo A, Herman JG, Mao L, Lee DJ, Gabrielson E, Burger PC, Baylin SB and Sidransky D (1995) 5′CpG island methylation is associated with transcriptional silencing of the tumour suppressor *p16*/*CDKN2*/*MTS1* in human cancers. *Nature Med* **1**: 686–692

Minth CD and Dixon JE (1990) Expression of the human neuropeptide Y gene. *J Biol Chem* **265**: 12933–12939

Monk M (1990) Changes in DNA methylation during mouse embryonic development in relation to X-chromosome activity and imprinting. *Philos Trans R Soc Lond B Biol Sci* **326**: 299–312

Riggs AD (1975) X-inactivation, differentiation and DNA methylation. *Cytogenet Cell Genet* **14**: 9–25

Silke J, Rother KI, Georgiev O, Schaffner W and Matsuo K (1995) Complex demethylation patterns at Sp1 binding sites in F9 embryonal carcinoma cells. *FEBS Lett* **370**: 170–174

Smith SS, Lingeman RG and Kaplan BE (1992) Recognition of foldback DNA by the human DNA (cytosine-5-)-methyltransferase. *Biochemistry* **31**: 850–854

Snibson KJ, Woodcock DM, Orian JM, Brandon MR and Adams TE (1995) Methylation and expression of a metallothionein promoter ovine growth hormone fusion gene (MT0GH1) in transgenic mice. *Transgenic Res* **4**: 114–122

Strauss BS (1991) The ‘A rule’ of mutagen specificity: a consequence of DNA polymerase bypass of non-instructional lesions? *Bioessays* **13**: 79–84

Szyf M (1994) DNA methylation properties: consequences for pharmacology. *Trends Pharmacol Sci* **15**: 233–238

Thiesen H-J and Bach C (1990) Target detection assay (TDA): a versatile procedure to determine DNA binding sites as demonstrated on SP1 protein. *Nucleic Acids Res* **18**: 3203–3209

Toth M, Müller U and Doerfler W (1990) Establishment of *de novo* DNA methylation patterns: transcription factor binding and deoxycytidine methylation at CpG anf non-CpG sequences in an integrated adenovirus promoter. *J Mol Biol* **214**: 673–683

Tucker KL, Beard C, Dausman J, Jackson-Grusby L, Laird PW, Lei H, Li E and Jaenisch R (1996) Germline passage is required for establishment of methylation and expression of imprinted but not of nonimprinted genes. *Genes Dev* **10**: 1008–1020

Vogelstein B, Fearon ER, Hamilton SR, Preisinger AC, Willard HF, Michelson AM, Riggs AD and Orkin SH (1987) Clonal analysis using recombinant DNA probes from the X-chromosome. *Cancer Res* **47**: 4806–4813

Woodcock D, Crowther P and Diver W (1987) The majority of methylated deoxycytidines in human DNA are not in the CpG dinucleotide. *Biochem Biophys Res Commun* **145**: 888–894

Woodcock DM, Lawler CB, Linsenmeyer ME and Warren WD (1997) Asymmetric methylation in the hyper-methylated CpG promoter region of the human L1 retrotransposon. *J Biol Chem* **272**: 7810–7816

Woodcock DM, Linsenmeyer ME and Warren WD (1998) DNA methylation in mouse A-repeats in DNA methyltransferase-knockout ES cells and in normal cells determined by bisulphite genomic sequencing. *Gene* **206**: 63–67

 *© Cancer Research Campaign 1999*
